# Adapting Bidirectional Encoder Representations from Transformers (BERT) to Assess Clinical Semantic Textual Similarity: Algorithm Development and Validation Study

**DOI:** 10.2196/22795

**Published:** 2021-02-03

**Authors:** Klaus Kades, Jan Sellner, Gregor Koehler, Peter M Full, T Y Emmy Lai, Jens Kleesiek, Klaus H Maier-Hein

**Affiliations:** 1 German Cancer Research Center (DKFZ) Heidelberg Germany; 2 Partner Site Heidelberg German Cancer Consortium (DKTK) Heidelberg Germany; 3 Helmholtz Information and Data Science School for Health Karlsruhe/Heidelberg Germany; 4 Heidelberg University Heidelberg Germany; 5 Hochschule Mannheim University of Applied Sciences Mannheim Germany; 6 Institute for Artificial Intelligence in Medicine (IKIM) University Medicine Essen Essen Germany

**Keywords:** Natural Language Processing, semantic textual similarity, National NLP Clinical Challenges, clinical text mining

## Abstract

**Background:**

Natural Language Understanding enables automatic extraction of relevant information from clinical text data, which are acquired every day in hospitals. In 2018, the language model Bidirectional Encoder Representations from Transformers (BERT) was introduced, generating new state-of-the-art results on several downstream tasks. The National NLP Clinical Challenges (n2c2) is an initiative that strives to tackle such downstream tasks on domain-specific clinical data. In this paper, we present the results of our participation in the 2019 n2c2 and related work completed thereafter.

**Objective:**

The objective of this study was to optimally leverage BERT for the task of assessing the semantic textual similarity of clinical text data.

**Methods:**

We used BERT as an initial baseline and analyzed the results, which we used as a starting point to develop 3 different approaches where we (1) added additional, handcrafted sentence similarity features to the classifier token of BERT and combined the results with more features in multiple regression estimators, (2) incorporated a built-in ensembling method, *M*-Heads, into BERT by duplicating the regression head and applying an adapted training strategy to facilitate the focus of the heads on different input patterns of the medical sentences, and (3) developed a graph-based similarity approach for medications, which allows extrapolating similarities across known entities from the training set. The approaches were evaluated with the Pearson correlation coefficient between the predicted scores and ground truth of the official training and test dataset.

**Results:**

We improved the performance of BERT on the test dataset from a Pearson correlation coefficient of 0.859 to 0.883 using a combination of the M-Heads method and the graph-based similarity approach. We also show differences between the test and training dataset and how the two datasets influenced the results.

**Conclusions:**

We found that using a graph-based similarity approach has the potential to extrapolate domain specific knowledge to unseen sentences. We observed that it is easily possible to obtain deceptive results from the test dataset, especially when the distribution of the data samples is different between training and test datasets.

## Introduction

Every day, hospitals acquire large amounts of textual data which contain valuable information for medical decision processes, research projects, and many other medical applications [1]. However, the huge quantity of reports is unsuitable for manual examination, and automatic access is hindered by the unstructured nature of the data [2]. Natural Language Understanding can help to tackle this problem by automatically extracting relevant information from textual data [3,4]. In this paper, we will focus on a subtask of Natural Language Understanding called Semantic Textual Similarity, which evolved within Natural Language Understanding as a dedicated research question aiming to address tasks like question answering, semantic information retrieval, and text summarization [5-9].

In the clinical domain, Semantic Textual Similarity has the potential to ease clinical decision processes (eg, by highlighting crucial text snippets in a report), query databases for similar reports, assess the quality of reports, or be used in question answering applications [1]. Furthermore, clinical reports are often of poor quality due to time limitations or due to the fact that many text snippets are simply copy-pasted from other reports [10,11]. This introduces low-quality data samples that make it harder for Natural Language Understanding algorithms to extract relevant information. In this context, Semantic Textual Similarity can be a key processing step when dealing with redundant text snippets [2].

State-of-the-art Natural Language Processing (NLP) methods for assessing the Semantic Textual Similarity of nonclinical data are developed and benchmarked based on the Semantic Textual Similarity benchmark, which compromises the SemEval Semantic Textual Similarity tasks from 2012 to 2017 [5] and is part of the General Language Understanding Evaluation dataset. In order to strengthen the development of Natural Language Processing tools for clinical and biomedical text data, which are often not publicly available, the team of the National NLP Clinical Challenges (n2c2), formerly known as i2b2 NLP Shared Tasks, has issued several tasks and organized challenges since 2006. This paper reports our participation in track 1, “n2c2/OHNLP Track on Clinical Semantic Textual Similarity,” of the 2019 National NLP Clinical Challenges. We present the 3 submitted systems, a further best performing variation of the different approaches, and a statistical analysis of the dataset. The aim of the track in which we participated was to predict the Semantic Textual Similarity between two clinical sentences. A similar task was already tackled in the BioCreative/OHNLP 2018 ClinicalSTS track [3,4].

The winners of Track 1: ‘n2c2/OHNLP 2018 Track on Clinical Semantic Textual Similarity’ [3] proposed 4 systems that combined string, entity, and number similarity features with deep learning features. In their best performing system, the winners trained a ridge regression model based on the prediction score of 8 independently trained models [3,12].  The second best performing team proposed an approach using Attention-Based Convolutional Neural Networks and Bidirectional Long Short Term Memory networks [3].

In recent years, the general Natural Language Processing domain made a huge step forward with the breakthrough of transfer learning which allows leveraging semantic knowledge from huge amounts of unlabeled text data. That is, a model can be pretrained on enormous unlabeled text data with multiple unsupervised tasks. The trained model captures a universal language representation and can be effectively fine-tuned on different downstream tasks. For example, the 2018 language model, Bidirectional Encoder Representations from Transformers (BERT), introduced a multilayer bidirectional Transformer that is trained on a massive amount of text in two unsupervised tasks: (1) next sentence prediction and (2) masked word prediction. To use the model for further downstream tasks, it is usually enough to add a linear layer on top of the pretrained model to achieve state-of-the-art performance for the desired downstream tasks [13]. Since the introduction of the Transformer and BERT, new variations of the original models perform even better by (1) introducing more pretraining tasks [14-16], (2) employing multitask learning approaches [17], and (3) combining the aforementioned approaches [18].

The application of pretrained models like BERT on clinical data comes with the question if the model can handle domain-specific nuances. One proposed approach to handling domain-specific nuances is to use transfer learning to adapt the model to clinical data [19-21]. Another approach is to incorporate already existing methods. To investigate the extent to which BERT can handle domain-specific nuances, we examined the performance of BERT on subgroups of sentences and found that it performed modestly with sentence types that were simply structured and highly specific (eg, sentences which prescribe medications). Based on these findings, we created 3 approaches with the aim to address the diversity of clinical sentences present in the given data.

**Figure 1:**
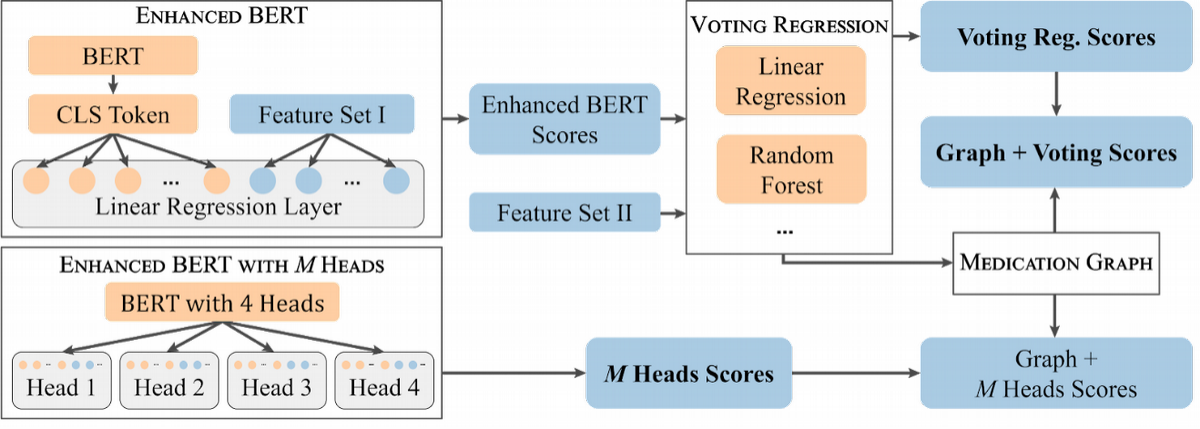
Overview of our pipeline for the different approaches. Blue boxes denote feature sets (see Multimedia Appendix 3) or scores which are also used as features, and the framed boxes denote processing steps. Bold scores correspond to our three submissions. [CLS] represents a classifier token which is used by BERT for sentence level downstream tasks [13]. The medication graph works only on a subset of the data with input from the Voting Regression. New scores are predicted and replaced with other scores only for this subset. BERT: Bidirectional Encoder Representations from Transformers.

To summarize our contributions (see Figure 1), we show that the use of BERT on clinical data can be enriched by the following:

- a simple modification of the BERT architecture by adding additional similarity features and employing a built-in ensembling method.
- a graph-based similarity approach for a subset of structured sentences in which the knowledge of the training set is extrapolated to unseen sentence pairs of the test set.

Additionally, we show that statistically analyzing the data reveals differences between the training and test datasets. This analysis made the process of interpreting the results easier.

The code to reproduce the results of this paper is available online [22].

## Methods

### Overview

Our methods were developed and tested on the data of the ClinicalSTS shared task, which consists of a collection of electronic health records from the Mayo Clinic’s dataset [4]. In total, 2054 sentence pairs were independently annotated to their degree of semantic textual similarity on a scale from 0 (not similar at all) to 5 (completely similar) by two medical experts. The focus of semantic textual similarity is whether two sentences have similar meaning and content in contrast to, for example, the number of words used in both sentences [3,4]. The created annotations are a mixture of integer and noninteger values, whereby the latter arise when averaging the result of multiple annotations. The training set consists of 1642 sentence pairs and the test set of 412. The performance of the different methods is measured by the Pearson correlation coefficient which aims to measure the linear correlation between the predicted similarity scores and the annotated similarity scores. More detailed information about the creation of the dataset, its properties, and its evaluation can be found elsewhere [1,3,4].

**Figure 2:**
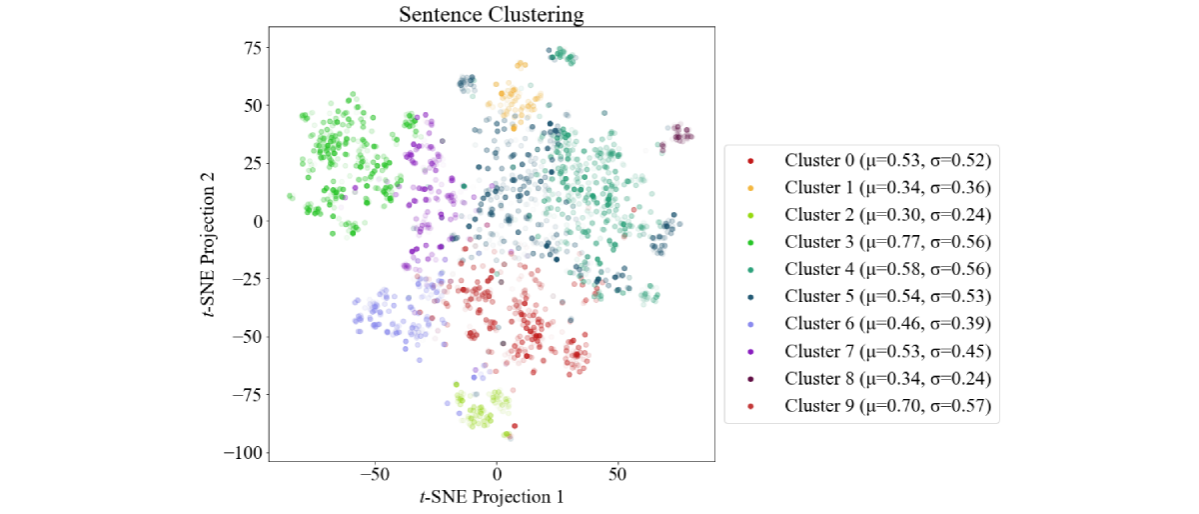
Clustering of the sentences to reveal BERT’s weaknesses. Each point represents a sentence pair from the training set, and the corresponding absolute score difference is visualized as opacity, or, in other words, the more opaque a point is, the higher the deviation from the ground truth. The points are the t-SNE projected InferSent embeddings of all sentence pairs. For each cluster, the average absolute deviation from the ground truth as well as the distribution of the differences is shown in the legend. Best viewed in colour. BERT: Bidirectional Encoder Representations from Transformers. t-SNE: t-distributed stochastic neighbor embedding.

**Figure 3:**
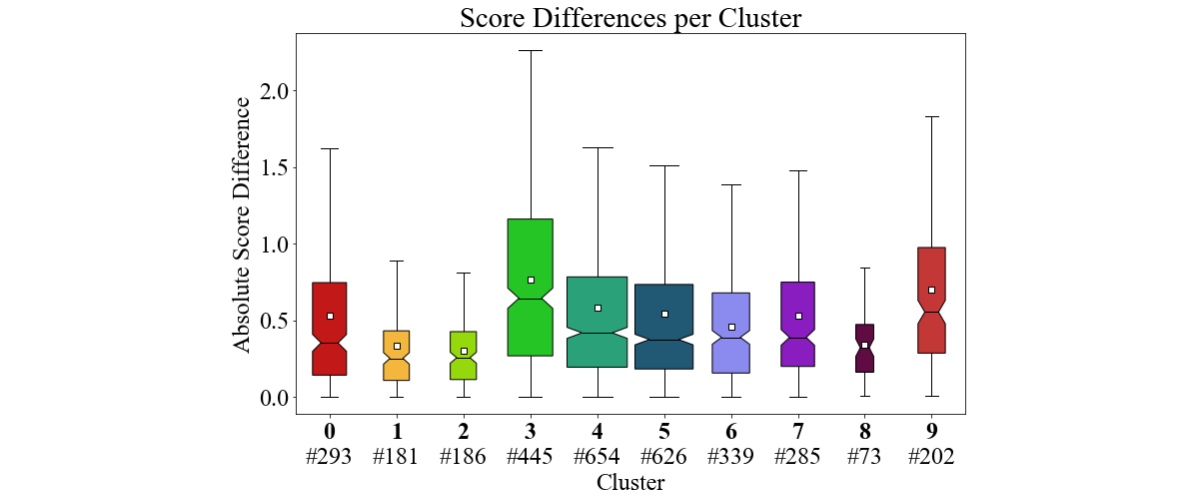
Box plot showing the absolute score differences for each cluster emphasizing the opacity information from Figure 2. The number below the bold cluster index is the cluster size. For each box plot, the following information is depicted: the box ranges from the lower to the upper quartiles with the notch at the median position. The whiskers extend up to 1.5 times the interquartile range. Remaining points (outliers) are not shown. The white square denotes the mean value.

We started by applying ClinicalBERT [23] to the dataset to obtain a baseline. That is, we used the [CLS] token from the last layer of the BERT model and fed its values to an additional linear layer that consists of a single neuron performing the similarity regression task. The whole network, including BERT and the additional layer, were trained on the Mean Square Error. We use the [CLS] token because it is designed for sentence classification and regression tasks. During training of BERT, [CLS] tokens are used for the next sentence prediction task. The [CLS] token is part of every sentence pair and captures the aggregated attention weights from each token of the sentence pair [13]. Next, we analyzed the predictions of BERT to find sentences with a high deviation from the ground truth. For this, we extracted InferSent embeddings for each sentence pair, as they are suited to cover the semantic representation of sentences [24], clustered them via -means, and calculated the absolute difference between the BERT scores and the ground truth for the whole cluster. The cluster analysis is shown in Figure 2. To make the comparison between the clusters easier, we show an overview of the absolute score differences per cluster in Figure 3. From these visualizations, we see that cluster 3 has the highest difference on average or, in other words, that BERT cannot handle these sentences well. Looking at the sentences, we see that this cluster is dominated by sentences which prescribe medication, for example  “ondansetron [ZOFRAN] 4 mg tablet 1 tablet by mouth three times a day as needed” or “furosemide [LASIX] 40 mg tablet 1 tablet by mouth two times a day.” This weakness was essentially the motivation for our third approach (medication graph) which focuses solely on the medication sentence type.

In the following section, we describe the approaches shown in our pipeline (Figure 1) in more detail. Information about the preprocessing steps and further implementation details can be found in Multimedia Appendix 1 and Multimedia Appendix 2.

### Approaches

#### Approach 1: Enhancing BERT With Features Based on Similarity Measures

The motivation behind this approach is to enhance BERT with additional information that BERT might not be able to capture in its model. On a token level, BERT uses a predefined tokenizer based on a set of rules; however, it might be valuable to compare arbitrary tokens based on character -grams. On a sentence-level, BERT does have a classifier token, [CLS], to compare two sentences. However, the [CLS] token was not designed to be a sentence-embedding [25,26]. Therefore, comparing embeddings like InferSent. which are specifically designed to represent the semantic of a whole sentence, might add additional valuable information to predict the similarity between two sentences.

In this approach, we used two kinds of similarity measures: (1) token-based and (2) sentence embedding–based. For a token-based similarity measure, -grams of characters are created and then compared with each other. For example, Jaccard Similarity compares the proportion between the intersection and the union of -grams in two input sentences. For a sentence embedding-based similarity measure, the embeddings of two sentences are compared, for example by taking the cosine similarity between the two embeddings of the two input sentences. The similarity measures were inspired by Chen et al [12].

We combined BERT with two feature sets of similarity measures at two different positions in our pipeline (Figure 1). In a first step named Enhanced BERT, we enhanced the [CLS] token of BERT with similarity measures from the first feature set (Feature Set I) before feeding the concatenated vector to the final linear regression layer. In a second step named Voting Regression, we fed the predicted output scores from Enhanced BERT together with a second feature set (Feature Set II) into several estimators (see Multimedia Appendix 3) whose predicted output scores were averaged with the help of a voting regressor [27]. Feature Sets I and II were created by successively trying out different combinations of similarity features in order to gauge the best performance. A breakdown of the two features sets can be found in Multimedia Appendix 3.

#### Approach 2: M-Heads

Ensembling methods have been very popular in recent machine learning challenges [28,29]. The general approach is to duplicate a model or parts of a model, repeat the prediction for each model, and aggregate the prediction results. The intuition is that different models can focus on various aspects of the input data (eg, different sentence types) so that they produce different predictions. The aggregation over these predictions can help to emphasize the group opinion over the dominance of a single model, thereby mitigating the risk that a model just reacted to noise in the input data [30].

We took up this point and decided to include a simple ensembling method directly into the architecture of BERT. More concretely, we duplicated the final linear layer (the head) which receives the last [CLS] token from the BERT model and which is responsible for calculating the regression (score prediction). We initialized each head layer with different weights to allow the different solutions per head. We employed a loss scaling which enforces specialization of the different heads similar to methods seen in other research [31,32]. A detailed description of our *M*-Heads updated scheme during training and how we performed predictions on new samples can be found in the Multimedia Appendix 4.

#### Approach 3: Medication Graph

In this approach, we focused on a subset of the sentence pairs which we named “medication sentences,” for example “ibuprofen 150 mg tablet 2 tablets by mouth every 7 hours as needed.” Further examples are listed in the discussion. These sentences are fairly structured and can be compared by analyzing individual entities. We used the MedEx-UIMA system [33,34] to extract medication related fields from the sentences and decided to use the entity’s active agent (“ibuprofen”), strength (“150 mg”), dose (“2 tablets”), and frequency (“7”). We considered the active agent as the major contributing factor in terms of the similarity of medication sentences. Hence, we modeled similarities between active agents that were then further modified by the remaining entities to retrieve a similarity score for each medication sentence pair.

Our general idea was to determine the property of similarity between active agent pairs as compared to unknown active agent pairs. That is, we assumed that the similarity of active agents A and B as well as B and C also contained information about the similarity between A and C. We generalized this process by constructing a graph containing all active agents as nodes with corresponding similarities assigned to the edges, using the shortest path between arbitrary active agents as a foundation to predict a similarity score which could then be further modified by the remaining entities (ie, every entity except the active agent).

In the following section, we describe how we delt with the remaining entities, in which way we constructed the graph of all active agents, and how we used this information to predict similarity scores for new sentence pairs.

### Feature Construction

Even though we considered the active agents as the central part regarding sentence similarity, we still did not want to neglect other influences and, hence, we constructed a set of additional features per sentence pair, which reflect the similarity of everything except the active agents. More concretely, we constructed a set of similarity features Δ_k_ and compared the entity value of the first sentence *e*_k,1_ with the entity value of the second sentence *e*_k,2_. For nominally scaled entities, we calculated Δ_k_ as

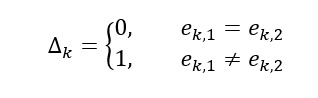



and for ratio-scaled entity types, we used the squared difference

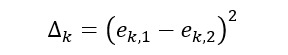



For entities like “strength” (eg, “4 mg”), we first separated the unit (“mg”) from the number (“4”), used the nominal approach to compare the unit, and applied the squared difference equation on the number part. This differentiation gives us k=1 , … , *N* features per sentence in total (N=5 in our case, since we used strength with amount and unit, and dose with amount and unit as well as frequency).

### Graph Construction

We used all medication sentences S = (*a_1_, a_2_, s*, Δ_1,_ …, Δ*_N_*) from the training set with the active agents *a_1_* and *a_2_* from the sentence pair, the similarity score *s*, and the remaining entity features Δ_k_. We constructed our similarity graph *G* (*V, E*) by using the possible active agents *A_i_* as nodes *V=*{*A*_1_, *A*_2_*,* …} and connected all node pairs which occurred together in a sentence pair. More precisely, we constructed an edge set *E={( A_i_*, *A_j_, w_ij_*)_(_*_i,j_*_)ϵ_*_P_* with the set of all possible active agent pairs and the edge weight

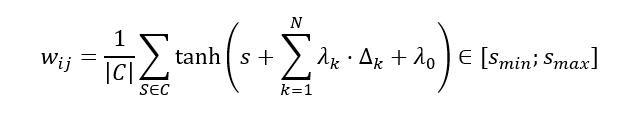



which models the modified similarity score *w_ij_* between the active agents. *C* denotes the set of all sentence pairs with the same active agents, and λ represents weights for the entity differences learned during the training process (see later text). λ_0_ can be interpreted as a bias. The tahn(*x*) function limits the change of the similarity score, *s*, and the final result is clipped to stay in the valid range defined by *s*_min_ = 0 and *s_max_ = 5*.

The intuition here is that the weights, *w_ij_*, should model the similarities between the active agents without factoring in the remaining entities. However, the true similarity value is not available as the similarity score, *s*, is also influenced by the remaining entities. The idea is that the weighted sum between the weights, *w_ij_*, and the differences, Δ*_k_*, allows us to alter the similarity score, *s*, in a way so that *w_ij_* models the true similarity between the active agents. The outer sum, responsible for averaging the items in the set *C*, is only necessary because it is possible that multiple sentence pairs with the same active agents exist.

### Inference

The goal of the inference phase was to calculate a sentence similarity score, *s*, between two active agents, *A_i_* and *A_j_,* based on a similarity score, 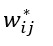 , obtained from the graph and the entity differences, Δ*_k_*. This consisted of two steps: first, we calculated the active agent similarity via the graph, and then we altered this similarity to account for the remaining entity features Δ*_k_.*

Step 1: In its simplest form, the similarity between two active agents is just the weight of the edge between the two corresponding active agent nodes. For example, *w_ij_* = 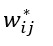. However, this is only possible when the weight already occurs in the training set and is not applicable in general, as *G* (*V, E*) is not a complete graph, and it may be the case that an edge between the two active agent nodes does not exist. As we still wanted to make a prediction for these cases, we proposed to find the shortest path between these two active agent nodes and aggregate all edge weights along the way. This assumes a transitive relationship between the nodes or, for example, when there is a connection between *A*_1_ and *A*_2_ as well as *A*_2_ and *A*_3_, we can still say something about the nonexisting connection between *A*_1_ and *A*_3_. More concretely, we aggregate the information 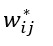 along the shortest path

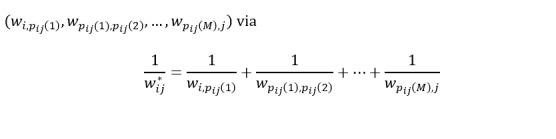



where *p_ij_*(1), *p_ij_*(2), …, *p_ij_*(M) denote the indices of the nodes on the shortest path between *A_i_* and *A_j_*. This equation resembles the formula for calculating the resistance of parallel circuits

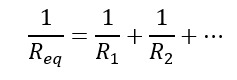



with the final resistance, *R_eq_*, of the circuit and the resistances, *R_i_*, of the individual flows. We chose this formula because the final resistance, *R_eq_*, is always smaller than the individual resistances, *R_i._* For instance, *R_eq_* ≤ min (*R*_1_, *R*_2,_ …)

[35]. In our case, this implies that the score 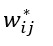 obtained from the graph is always lower than any of the scores along the shortest path. This relies on our assumption that it is not possible to restore dissimilarities; for example, if there is already a score of 1 (low similarity) on an edge, we do not want to increase this value further by adding more connections, as we already know that at least two active agents are dissimilar.

Step 2: The weight, 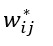, is the prediction for the similarity of an active agent pair. The final goal was to retrieve a prediction score, *s*, for a sentence pair which is also influenced by the remaining entity features, Δ*_k_*. We accounted for this by altering the predicted score again by

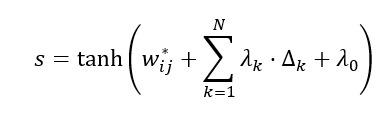



to retrieve a similarity, *s*, for the sentence pair.

**Figure 4:**
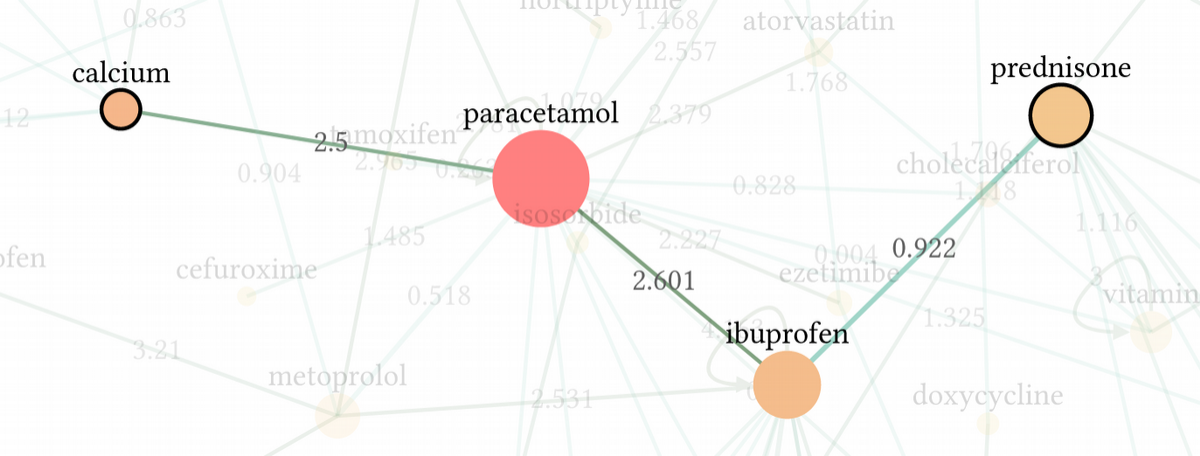
Excerpt of the medication graph, which models similarities between active agent pairs. On the edges, the modified similarity score, <inline-graphic xlink:href="medinform_v9i1e22795_fig10.png" mimetype="image" xlink:type="simple"/>, is shown. The full graph is available as an online widget, which provides further information and shows the graph calculations between arbitrary active agent nodes.

Figure 4 shows an excerpt of the graph, which uses all the sentence pairs in the training set. The shortest path between the active agents “calcium” and “prednisone” is highlighted to visualize the prediction steps. Detailed calculations are available in the online version of the graph [36].

It may be possible that the sentences contain additional information that we do not cover in our approach, such as additional words, the relation between words, etc. For this reason, we combined the similarity score, *s_g_*, from the graph with the BERT scores, *s_b_*, in a Support Vector Regressor trained on all sentences in the training set to retrieve a final prediction score. We used Radial Basis Functions as kernel and optimized the regularization parameter *C* as well as ε (ε-tube without penalty) during the learning process of λ*_k_*.

### Learning λ*_k_*

The parameters λ*_k_* are responsible for the transformation between the active agent and sentence similarities as they reflect the importance of each entity feature, which also includes scaling differences. We did not manually craft these weights but learned them in a random walk process instead. The general idea was to randomly change the weights and see whether this improved the graph performance and, only if it did, we kept the change.

For a more stable evaluation, we split the training data into 10 folds, built a graph based on each training set, and evaluated the graph performance based on the corresponding test set. For evaluation, we calculated the mean squared error between the prediction scores and the ground truth. We did not use the Pearson correlation coefficient here because the correlation on a subset may not be as helpful for the correlation on the complete dataset as a measure which directly enforces a closeness with the ground truth.

Let λ = (λ_0,_ λ_1,_ …, λ*_N_*) denote the vector with the current value of the weights (randomly initialized in the beginning) and let MES(λ) denote the error when using these weights with the predictions from all folds. Then, we randomly selected an index, *k*, and altered the corresponding weight

λ'*_k_* = λ*_k_* + *Ν*(0,1)

via a sample from a standard normal distribution so that we obtained a new weight vector

**λ’**= (λ_0,_ λ_1,_ …, λ'*_k_*, …, λ*_N_*)

which we evaluated again on the graphs from all folds, keeping the change if

MES(λ’) < MES(λ)

We repeated this process in two iterations, alternating with the process of hyperparameter tuning of the SVR model, until we observed no further improvements. For each random walk process, we applied 50 update steps. During development, we found that this setting was already sufficient and that the resulting weights tended to remain unchanged after these updates. For the SVR model, we applied a grid search to find values for the hyperparameters *C* and ε-tube. We used the final weights to construct a new graph (based on all training data) used to predict the similarity of new sentences.

## Results

### Dataset Evaluation

**Figure 5:**
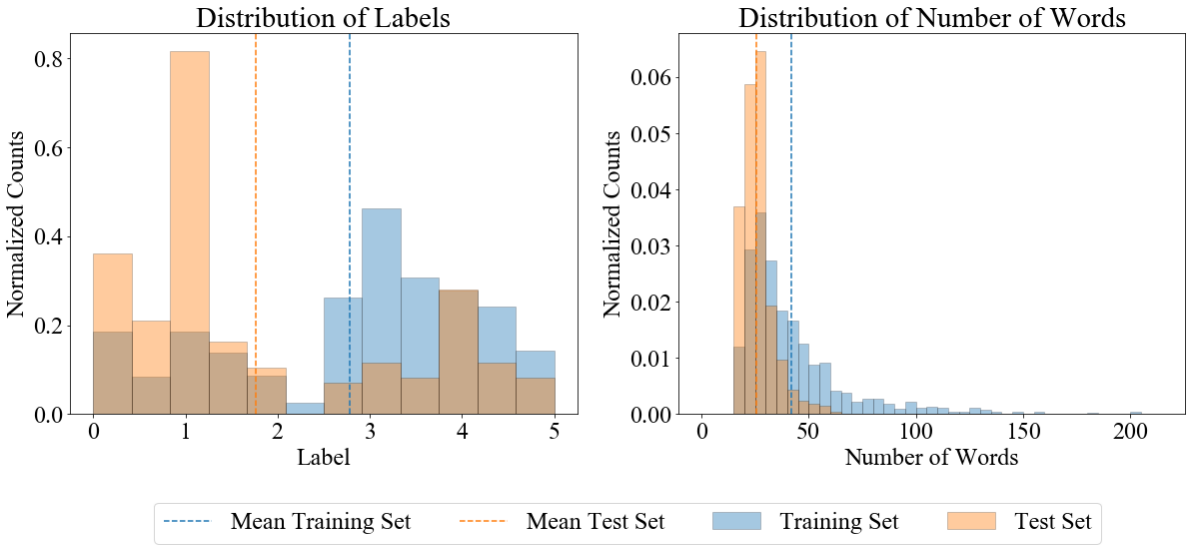
Histogram of the label distribution and word lengths of the training and test set.

In order to help with the interpretation of our results in the next section, we applied some basic statistical analysis on the training and test set, which revealed some imbalances. On average, the similarity score of the sentences in the training set (approximately 2.79) was higher than in the test set (approximately 1.76), whereas the standard deviation was slightly higher in the test set (approximately 1.52) than in the training set (approximately 1.39). This is also indicated by the left histogram chart of Figure 5, which, for example, reveals that sentence pairs with a score of approximately 1 are the most prominent ones in the test set, whereas in the training set, sentence pairs with a score of approximately 3 occur most often.

The right histogram chart of Figure 5 shows the distribution of the number of words of the training and test data. On average, the sentences in the test set tended to be shorter than in the training set, with an average sentence length of approximately 26 words per sentence pair (SD of approximately 7 words) in the former and approximately 42 words per sentence pair (SD of approximately 26 words) in the latter.

**Figure 6:**
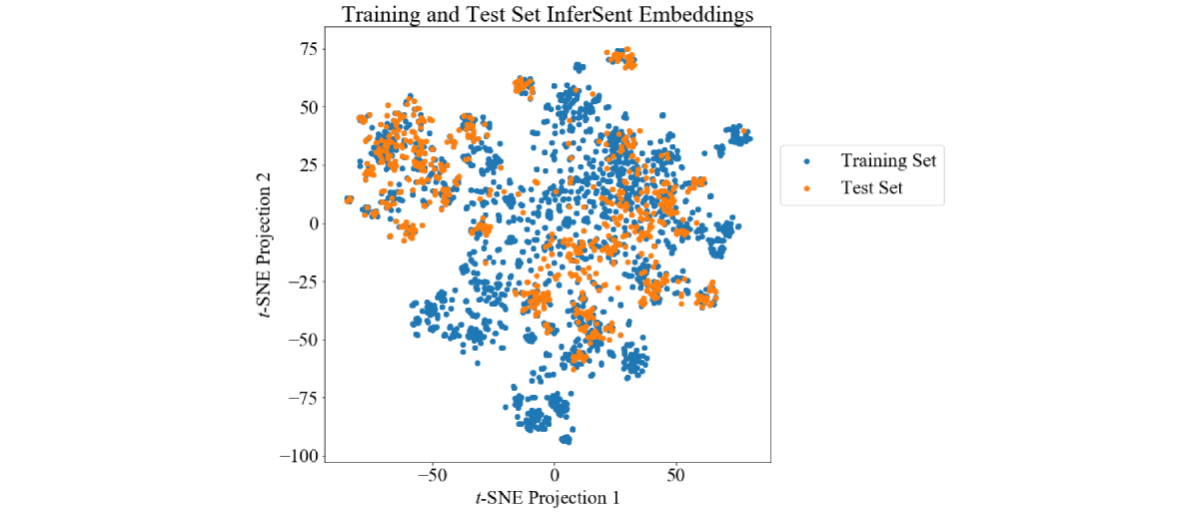
t-SNE projected InferSent embeddings of the sentences in the training and test dataset. Different groups of points correspond to different sentence types. For example, the group on the left upper side corresponds to the medication sentences. t-SNE: t-distributed stochastic neighbor embedding.

Finally, we calculated InferSent embeddings of the sentences in the training and test dataset and visualized them in a t-SNE (t-distributed stochastic neighbor embedding) plot (Figure 6). This shows that the sentence types occurring in the test dataset represent only a subset of those occurring in the training dataset, with many clusters of the training set being unoccupied by the test set, such as the blue cluster in the bottom of Figure 6 without sentences of the test set in the neighborhood.

### Evaluation Results

We evaluated all runs on 3 different sets. Firstly, we used the training set with *k* = 150 folds to reduce the influence of noise in the data, to increase the comparability of our models, and to easily employ another ensembling technique for the test set. We wanted to measure the correlation of the training set and not of one of the folds to get comparable results. For this, we concatenated the predictions from each fold together and then calculated the Pearson correlation coefficient only once based on all scores. That is, we did not calculate a Pearson correlation coefficient for each fold, but rather collected the scores from all folds first. The consequence of this approach is that we cannot provide information about the variance, because only one Pearson correlation coefficient value is available.

Secondly, for the evaluation of the test set, we employed an additional ensembling technique by using the model for each fold to calculate a prediction for a sentence pair and then averaged all predictions.

Table 1 gives an overview of our results for the different datasets and approaches. Our best result with a Pearson correlation coefficient of 0.883 was achieved by combining enhanced BERT with *M*-Heads and the medication graph. For comparison, the winner of track 1 from IBM Research reached a Pearson correlation coefficient of 0.901 in their best submission [4].

**Table 1:** Summarization of the different approaches and their results. Training and test Pearson correlation coefficient scores are rounded to 3 decimal places.

Approach		Training set	Test set
**Approach 0: baseline**			
	ClinicalBERT	0.850	0.859
**Approach 1: voting regression**			
	Enhanced BERT	0.851	0.859
	Voting Regression	0.860	0.849^a^
**Approach 2: *M*-Heads**			
	Enhanced BERT with *M*-Heads	0.853	0.876^a,b^
	Enhanced BERT with *M*-Heads + Med. Graph	0.853	0.883^c^
**Approach 3: medication graph**			
	Voting Regression + Med. Graph	0.862	0.862^a^

^a^Our submissions.

^b^We submitted a score of 0.869 for this setting because we were able to use only 10 *k*-folds due to a shortage of time.

^c^Our best result of the test set.

## Discussion

Our 3 approaches performed differently on the two datasets. In the following sections, we discuss the results in more detail and give our thoughts.

### Approach 1: Voting Regression

Evaluating the pure ClinicalBERT model, we see that the Pearson correlation coefficient is slightly higher for the test set as compared to the training set. The Enhanced BERT architecture led to an almost neglectable improvement on the training set and, in the test set, to no improvement at all. This indicates that, in this case, the additional features do not provide more information than what is already contained in the [CLS] token from the last hidden layer of BERT.

The Voting Regression approach showed an improvement of the Pearson correlation coefficient of the training set; however, for the test set, the performance decreased. These results might be traced back to overfitting of the training set. However, the decrease in the test set might also be explained by the imbalances between the training and test set.

### Approach 2: M-Heads

Adding *M* = 4 heads to BERT increased the Pearson correlation coefficient of both the training and test set as compared to ClinicalBERT. Especially for the test set, this indicates that the combination of the different heads improves BERT’s performance.

### Approach 3: Medication Graph

Replacing the scores of the sentence subset which prescribes medications (cluster 3) with the medication graph scores led, in both cases (approaches 1 and 2), to an improvement for the test set. For the training set, however, we saw only marginal improvements, such as 0.860 to 0.862 from approach 1 to approach 3. This might be due to the Pearson correlation coefficient metric. In our experiments, we also evaluated our approaches with the Mean Squared Error between the predictions and the ground truth only on the subset of medication sentences. Without applying the medication graph (approach 1), we obtained a Mean Squared Error of 0.70, and with the medication graph (approach 3) a Mean Squared Error of 0.58. Combining the *M*-Heads approach with the medication graph yielded our best results for the test set, which indicates that BERT does have problems handling this domain-specific knowledge and therefore cannot cope well with these specific types of sentences.

Why did the medication graph perform better for the test set than for the training set? First, we observe that the test set contained more low-ranked sentences (see Figure 5); in particular, the medication sentences had lower scores. For the training set, the mean and standard deviation of the scores was 2.03 and 1.05, respectively, whereas the scores for the test set had only a mean and standard deviation of 1.10 and 0.50, respectively. We also noticed that the medication graph tended to dampen the prediction or, in other words, it led to lower scores. For example, the mean prediction score was 2.58 before and 1.78 after score replacement of the 94 medication sentences in the test set (see Table 2), which shows some example sentences of how the medication graph altered the scores). This could be due to two reasons: (1) the scores on the edges in the graph tended to be low (1.87 on average), and (2) the weight combination enforced low scores when there was at least one edge with a low score, which could explain why the medication graph achieved better predictions. This effect is facilitated by the fact that the training dataset contained only 147 out of 1642 (8.95%) of sentences that prescribed medication, whereas the test set contained 94 out of 412 (22.82 %) medication sentences.

**Table 2:** Comparison of the Pearson correlation coefficient scores predicted by the Voting Regression (approach 1, A1) and the medication graph (approach 3, A3) via randomly selected example sentences. T denotes the ground truth score for the corresponding sentence pair. This table shows only the relevant entities from the original example sentences.

Sentence a	Sentence b	T	A1	A3	Set
Ondansetron, 4 mg, 1 tablet, three times a day	Amoxicillin, 500 mg, 2 capsules, three times a day	3.0	1.68	1.70	Training
Prozac, 20 mg, 3 capsules, one time daily	Aleve, 220 mg, 1 tablet, two times a day	0.5	2.02	1.68	Training
Hydrochlorothiazide, 25 mg, one-half tablet, every morning	Ibuprofen, 600 mg, 1 tablet, four times a day	1.5	1.59	1.70	Training
Aleve, 220 mg, 1 tablet, two times a day	Acetaminophen, 500 mg, 2 tablets, three times a day	1.5	2.74	1.68	Test
Lisinopril, 10 mg, 2 tablets, one time daily	Naproxen, 500 mg, 1 tablet, two times a day	1.0	2.29	1.69	Test

### Conclusions

To tackle the problem of semantic textual similarity of medical data, we developed 3 different approaches. We proposed to add additional features to BERT and to weigh different regression models based on the BERT result and other features. Moreover, we proposed the application of *M*-Heads and an attempt to automatically extrapolate medical knowledge from the training data. We observed that the success of the different methods strongly depended on the underlying dataset. In future work, it might be interesting to evaluate the methods on different and bigger datasets from other domains. The medication graph could be a powerful method with the possibility to be applied to other domains where it is necessary to extrapolate information from known entities and where it is not possible to calculate this information directly. It may also be used to model other concepts which exist in the medical domain, such as ontologies.

## Appendix

Multimedia Appendix 1 Preprocessing.

Multimedia Appendix 2 Implementation Details.

Multimedia Appendix 3 Detailed description of Feature Set I and Feature Set II.

Multimedia Appendix 4 M-Heads: training and prediction.

## Abbreviations

BERT: Bidirectional Encoder Representations from Transformers
NLP: Natural language Processing
n2c2: National NLP Clinical Challenges
OHNLP: Open Health Natural Language Processing
