# Supplementary material for: Adapting Bidirectional Encoder Representations from Transformers (BERT) to Assess Clinical Semantic Textual Similarity: Algorithm Development and Validation Study
Source: JMIR Med Inform. 2021 Feb 3;9(2):e22795. doi: 10.2196/22795 (PMC7889424; doi:10.2196/22795)
Supplement: Multimedia Appendix 1 [file medinform_v9i2e22795_app1.docx]

## Multimedia Appendix 1. Preprocessing.

We developed a set of different preprocessing steps to unify and clean the dataset for our analysis. However, not every preprocessing step is suitable for each model so we first define a set of possible steps and later declare which steps we used for which model.

- ContractionExpander: we use the pycontraction Python package [1] for contraction expansion (eg, “we’ll” → “we will”) to normalize the text and reduce ambiguities.
- NumberUnifier: we convert all textual representations of numbers to the corresponding numerical literal (eg, “forty-two” → “42”) to unify the representation of words which convey the same meaning.
- SpellingCorrector: we correct common spelling mistakes in the dataset (eg, “refil” → “refill”).
- MedicationRemover: as preparation for our medication graph, we remove all vendor drug names from the medication sentences and keep only the general active agent name (e.g. “metoprolol succinate [TOPROL XL] 25 mg...” → “metoprolol succinate 25 mg...”).
- SentenceTokenizer: as a preliminary step before word-tokenization, we used the library segtok [2] to split the sentences. This library is optimized for European languages and employs a two-step approach. First, a sentence is split on common sentence markers (eg, “.,?”) and then every split is evaluated again by considering the surrounding and checking for false positives (eg, to handle cases like name initials correctly) [3].
- WordTokenizer: after the sentence tokenization, the word tokenizer from the library segtok is applied [3].
- PunctionRemover: we used a rule-based approach to remove punctuations.
- LowerCaseTransformer: this processing step transfers uppercase letters to lowercase letters.
- StopWordsRemover: we removed common stop words as well as task-specific words like “tablet” or “medication” due to their high frequency and therefore lower significance for the used similarity measures.
- Lemmatizer: we used the Python library pattern to lemmatize the words, i.e. to normalize words to its root (eg, “moved steadily” → “move steadily”) [4].

Before we used BERT, the preprocessing steps ContractionExpander, NumberUnifier, SpellingCorrecter and LowerCaseTransformer were applied to the dataset. For Feature Set I and II, all mentioned preprocessing steps except the MedicationRemover were applied.

### References

1. GitHub. ian-beaver/pycontractions, https://github.com/ian-beaver/pycontractions.
2. GitHub. fnl/segtok, https://github.com/fnl/segtok.
3. F. L. segtok - a segmentation and tokenization library. 2015 January 12.
4. Smedt TD, Daelemans W. Pattern for Python. Journal of Machine Learning Research. 2012;13(66):2063-7.
