# Supplementary material for: Adapting Bidirectional Encoder Representations from Transformers (BERT) to Assess Clinical Semantic Textual Similarity: Algorithm Development and Validation Study
Source: JMIR Med Inform. 2021 Feb 3;9(2):e22795. doi: 10.2196/22795 (PMC7889424; doi:10.2196/22795)
Supplement: Multimedia Appendix 2 [file medinform_v9i2e22795_app2.docx]

## Multimedia Appendix 2. Implementation Details.

For all approaches, we used the PyTorch Transformers library [1] for all BERT related calculations. As a model, we used ClinicalBERT (biobert_pretrain_output_all_notes_150000) [2] which builds upon BioBERT [3]. The training of Enhanced BERT and *M*-Heads was done with 10 epochs, a maximal sequence length of 128 and a learning rate of $2^{-5}$.

### References

1. Wolf T, Debut L, Sanh V, Chaumond J, Delangue C, Moi A, et al. HuggingFace's Transformers: State-of-the-art Natural Language Processing. arXiv:191003771 [cs]. 2020 Feb, 11.
2. Alsentzer E, Murphy J, Boag W, Weng W-H, Jindi D, Naumann T, et al., editors. Publicly Available Clinical BERT Embeddings. 2019 2019-06. Minneapolis, Minnesota, USA: Association for Computational Linguistics.
3. Lee J, Yoon W, Kim S, Kim D, Kim S, So CH, et al. BioBERT: a pre-trained biomedical language representation model for biomedical text mining. Bioinformatics. 2020 2020/02/15;36(4):1234-40. doi: 10.1093/bioinformatics/btz682.
