# Supplementary material for: Adapting Bidirectional Encoder Representations from Transformers (BERT) to Assess Clinical Semantic Textual Similarity: Algorithm Development and Validation Study
Source: JMIR Med Inform. 2021 Feb 3;9(2):e22795. doi: 10.2196/22795 (PMC7889424; doi:10.2196/22795)
Supplement: Multimedia Appendix 3 [file medinform_v9i2e22795_app3.docx]

## Multimedia Appendix 3. Detailed description of Feature Set I and Features Set II.

Features Set I was directly added to the final linear regression layer of BERT and was created by empirically evaluating different combinations of similarity measures during development. It consists of the following token-based text distance measures with *n*-grams of $n=3$ (ie, we used three characters for comparison): Jaro and Jaro-Winkler distance, Sørensen–Dice coefficient, overlap coefficient and cosine similarity [1]. Moreover, we calculated InferSent2 sentence embeddings and a mean pooled sentence representation of the GloVe word embeddings (used model: glove.840B.300d [2]). For each embedding, we calculated different distance metrics (cosine similarity, Euclidean, Manhattan and the Minkoski distance) from the corresponding sentence pair.

Feature Set II was also obtained by comparing the overall performance of the model for different similarity features. It consists of text distance measures with *n*-grams of $n=3$ and $n=4$: Damerau-Levenshtein, Jaro-Winkler and the Bag distance as well as a square root based normal compressor distance (SqrtNCD). Furthermore, it includes the Manhattan, the Minkowski and the Euclidean as well as the cosine similarity based on an InferSent1 sentence embedding as well as a mean pooled sentence representation of the GloVe word embeddings. For the Voting Regression, we combined a set of linear regression models (least squares, lasso, epsilon-insensitive fitting, SVR) with ensembling models (random forest, AdaBoost, gradient tree boosting).

### References

1. GitHub. life4/TextDistance, https://github.com/life4/textdistance.
2. GitHub. stanfordnlp/GloVe, https://github.com/stanfordnlp/GloVe.
