# Supplementary material for: Adapting Bidirectional Encoder Representations from Transformers (BERT) to Assess Clinical Semantic Textual Similarity: Algorithm Development and Validation Study
Source: JMIR Med Inform. 2021 Feb 3;9(2):e22795. doi: 10.2196/22795 (PMC7889424; doi:10.2196/22795)
Supplement: Multimedia Appendix 4 [file medinform_v9i2e22795_app4.docx]

## Multimedia Appendix 4. *M*-Heads: Training and Prediction.

**Training**

Given an input consisting of a sentence pair, each of the $M$ heads calculates a similarity score $s_{h}$ based on the last [CLS] token. Then, we calculate the corresponding loss via ($s^{*}$ denotes the ground truth)

$$l_{h}=\mathrm{MSE}(s_{h},s^{*})$$

Now, the idea is to scale the individual losses in a way so that the head which is already closest to the input, i.e. the one with the lowest loss, gets updated most. For this, we first find the winning head

$$h^{*}=\underset{h=1,\ldots,M}{\mathrm{argmin}}\left( l_{h} \right)$$

and then calculate a weight for each loss

$$\alpha_{h}=\left\{ \begin{aligned} \beta_{1}, &h=h^{*} \\ \frac{\beta_{2}}{M-1}, &h\neq h^{*} \end{aligned} \right.$$

with $\beta_{1}>\beta_{2}$ (e.g. we used $\beta_{1}=0.95$ and $\beta_{2}=0.05$ in our experiments). Next, we scale each loss by its corresponding weight and reduce the losses to a scalar by averaging over the head losses

$$\mathcal{L=}\frac{1}{M}\sum_{h=1}^{M} {\alpha_{h}\cdot l}_{h}$$

This way, the winning head receives the highest weight $\beta_{1}$ so that its loss is considered most while the other heads have to share the remaining contribution $\beta_{2}$.

**Prediction**

To predict an output score $s$ for a new sentence pair, we simply ask each head for its prediction $s_{h}$ and then use the average

$$s=\frac{1}{M}\sum_{h=1}^{M} s_{h}$$

as a similarity score. We used $M=4$ heads for our built-in ensembling approach since this value performed best in our experiments.
